# Evolutionary Consequences of Male Driven Sexual Selection and Sex-Biased Fitness Modifications in *Drosophila melanogaster* and Members of the *simulans* Clade

**DOI:** 10.1155/2015/756269

**Published:** 2015-09-01

**Authors:** Santosh Jagadeeshan, Wilfried Haerty, Monika Moglinicka, Abha Ahuja, Scot De Vito, Rama S. Singh

**Affiliations:** Department of Biology, McMaster University, 1280 Main Street West, Hamilton, ON, Canada L8S 4K1

## Abstract

Males have evolved a variety of behavioral, morphological, and physiological traits to manipulate their mates in order to maximize their chances of success. These traits are bound to influence how females respond to male behaviors and influence the nature of sexual selection/conflict. A common consequence of aggressive male mating strategies in* Drosophila melanogaster* is the reduction of female lifespan. Our study shows that this is common across members of the* simulans *clade. Reduced life expectancy of females implies that female contribution to a population is less than that of males per generation. Fitness differences between the sexes in every generation will invariably affect overall population fitness. How natural selection responds to the female deaths and thereby the unequal fitness of the sexes has rarely been addressed. We shed light on this issue and provide evidence, which suggests that additional gains of fitness by males due to their longevity and continued mating may provide one explanation as to why the loss of female fitness may be “invisible” (effectively neutral) to natural selection. Male driven sexual selection and additional, transgenerational gains of male fitness can be an important force of evolutionary change and need to be tested with other organisms.

## 1. Introduction

In most sexually reproducing species, anisogamy presents a dichotomy in reproductive investments, where smaller and more numerous male gametes must typically compete for the fewer and larger female gametes. Males therefore typically compete for additional mating whereas females typically allocate their resources into offspring production and care [1]. This provides opportunities for more intense selection on males [1, 2], and male variations enhancing their own fitness will be under selection, even if it is costly to females. In such cases, sex specific selection can lead to accumulation of mutations beneficial to the males but detrimental to females [3–6]. As a result, sexual conflict arises due to differences in reproductive interests and investments between the sexes and can translate into differences in fitness optima between sexes for a given trait [5]. In addition, the nature of sexual conflict may depend on the extent to which one sex's action pushes the other away from its fitness optimum. It has been known for a while now that the very act of mating, in many cases, is often associated with some form of physical and/or physiological harm to females, as a result of male reproductive strategies [7, 8]. There is evidence of male induced harm to female which ranges from forced mating in flies, butterflies, fish, frogs, birds, and primates [9–12] to traumatic insemination in bedbugs [13]. Studies from fruitflies, butterflies, and worms show that even if males do not directly (physically) harm females, molecular components of the male ejaculate contribute to the reduction of female's lifespan [14–16]. These observations reveal that males have evolved a variety of means to increase their own fitness.

The widespread evidence of mating induced harm to females is compelling enough to expect, under the sexual antagonistic coevolution theory, that the most adversely affected sex (females) would counter-adapt to minimize the loss of fitness due to manipulations of the opposite sex [4, 17]. It would appear, however, that such expectations are not pervasive and depend not only on the extent of the costs and benefits of male manipulations, but also on the mechanisms available to female to counter respond (see [17] for a discussion). A better appreciation of this issue therefore requires a closer inspection of the fitness consequences of male and female life history [18]. In this paper we examine the sex specific costs of mating in closely related species of the* D. melanogaster* subgroup and further explore fitness contributions of the sexes within* D. melanogaster*, with a specific focus on male life history traits.

*Drosophila melanogaster* has played a key role as a model organism in which sexual conflict has been well studied [19]. In* D. melanogaster*, males are quite aggressive and persistent in their attempts to court females enhancing their chances of securing multiple mates [2, 20, 21]. However, female fecundity and lifespan are reduced as a result of the aggressive and persistent courtship attempts of males [22, 23]. At the postmating stage, seminal fluid proteins are used by males to gain precedence over sperm from other males for fertilization [24–26], effectively “transferring” a part of male-male competition to take place within the females' reproductive tract. Some of these seminal fluid proteins also influence female ovipositional behavior and physiology [27, 28], and the toxicity of some seminal fluid proteins involved in intrasexual competition also reduces a female's lifespan [29–32]. These negative effects of male reproductive life history on females are exacerbated in experimental manipulations where females are not allowed to coevolve with males [15]. On the other hand, experimentally enforcement monogamy not only alleviated the costs but also increased fitness of the flies [6, 33]. Although* D. melanogaster* females are known to remate, they do not appear to accrue much benefits of remating [34]. Costs of mating have also been explored to some extent in* D. simulans* [35], but absence of similar studies in* D. mauritiana* and* D. sechellia* precludes inferences of whether the pattern of mating costs is common across these closely related species [36].

In other insect species, despite any fecundity benefits that females may receive from mating multiply [35, 37, 38], their reduced lifespan compared to males has obvious implications on sex specific lifetime reproductive success. This difference has consequences on the fitness contributions of the sexes to population fitness. Essentially, since males typically live longer, they may stand to gain from additional mating and contribute relatively more to population fitness. By this measure, the fitness of the sexes is unequal in every generation. It is possible therefore, in the long run, that the continual lower fitness of one sex can impose a load on population fitness [39, 40]. Indeed, simulations [41, 42] and selection experiments [43] indicate that sexual conflict and sexually antagonistic selection can reduce overall population fitness, thereby imposing additional costs on sexual reproduction. Therefore, while sexual conflict may be responsible for the differential fitness optima of the sexes, the maintenance of such a system and the potential manifestation of sexually antagonistic coevolution may come into question, especially if the costs imposed by the manipulating sex outweigh any gains in the affected sex. One may expect that natural selection would favor a sexual system where both sexes contribute equally to population fitness [44] or should at least resolve the conflict to minimize the difference in fitness optima between the sexes. Therefore, how does natural selection deal with the reduction of female lifespan in every generation in* Drosophila*? With respect to population fitness, the loss of female fitness must either be invisible to natural selection or be compensated in some other way.

One possibility is that, despite the potential of sexual conflict, males may not be pushing females beyond their threshold fitness optima, and any loss of fitness does not significantly affect population fitness. In this case, even though the potential for sexually antagonistic coevolution may exist, the fitness differential is not sufficient enough to elicit counter adaptations by females [17, 45]. The other possibility is that population fitness lost due to female deaths may be compensated or minimized in other ways. Here, male life history may become important. Males not only initiate sexual interactions; they gain from multiple matings and they generally outlive females. As a consequence of reproductive longevity, males typically have more opportunities to mate compared to females. For instance, in species with overlapping generations like* Drosophila* or humans, not only can males of any generation mate with surviving females of their own generation, but, due to their longevity, they can mate with females of successive generation as well. By this reasoning, males have more opportunities for fitness gains and therefore make greater fitness contributions to overall population fitness, relative to females. Therefore we suspect that, despite any differences in fitness optima of the sexes in lifetime reproductive success, the overall population fitness may not be reduced beyond a threshold for natural selection to act against.

We illustrate our argument through mating experiments and fitness assays in* D. melanogaster, D. simulans, D. mauritiana,* and* D. sechellia* species complex. These members of the* melanogaster* subgroup have radically different life histories and ecology [36, 46] which may influence the nature of sexual selection [47]. We then conducted a series of assays in* D. melanogaster* in order to highlight the fitness consequences of mating for the sexes, with an emphasis on the consequences (costs imposed and fitness gained) of male life history. Our results show that (1) mating induced reduction of female lifespan is common across* D. melanogaster* and* simulans* group; (2) although exposure to multiple males is in general detrimental to females, it has little effect on males across species; and (3) quite interestingly, in our assays with* D. melanogaster*, older males were competitive with younger males with respect to mating success. We discuss these results in the light of male driven sexual selection and its relative importance in the evolution of male traits, particularly those that are used to manipulate their mates. Rather than proposing a mechanism for conflict resolution, our intention here is to bring to attention the importance of multiple ways in which males gain fitness compared to females and the observation that male reproductive longevity enables additional transgenerational contribution to population fitness.

## 2. Material and Methods

### 2.1. *Drosophila* Strains

We used individuals from an outbred laboratory* Drosophila melanogaster* strain that was established by crossing 6 different geographical strains [48].* D. simulans* (0251.2),* D. mauritiana* (0.248.1) strains were obtained from the Tucson* Drosophila* Stock Centre.* D. sechellia* strain was obtained from Dr. Jean R. David (CNRS, Gif sur Yvette, France). Flies were kept at 25°C on a standard cornmeal and molasses medium. Virgin females and males were collected at emergence under light CO_2_ anesthesia and housed separately and aged for 4–8 days on cornmeal and molasses medium. In experiments that involve several treatments and replicates, each treatment was done in separate vials that were carefully labeled and dated in order to facilitate tracking of flies through the experiments. In addition, flies were carefully aspirated into vials to ensure no injuries due to handling.

### 2.2. Effect of Male Density on Female Fertility and Longevity of Both Sexes

This experiment was designed to highlight the potential deleterious effects of increasing male density on both female and male longevity, as well as on female fertility. We performed the experiment on* D. melanogaster, D. simulans, D. mauritiana*, and* D. sechellia* to test for a potential species effect. Four different treatments consisting of one female housed with an increasing number of males (1, 3, 6, or 9 males) for her entire lifetime were initiated in separate vials. In all the treatments males were not renewed and therefore subject to ageing. A minimum of 20 replicates per species and treatments were initiated. Every 5 days, for each treatment, and until all females and males died, living females and males were counted and transferred into new vials. The old vials were retained and the number of progeny that emerged from each vial was recorded.

### 2.3. Effect of Varying Exposure to Males on Female Fertility and Longevity

The aim of this experiment was to assess how female longevity and fertility are affected by male exposure for brief periods of time in* D. melanogaster*. Three treatments and two controls were initiated. The treatments varied from each other in the frequency of female exposure to males. Treatment 1 consisted of females mated with males only once, and their exposure was restricted to only other mated females subsequently. Treatment 2 consisted of females mated once and exposed to males once every seven days during their entire lives. Treatment 3 consisted of females exposed to males every three days. Control 1 was composed of virgin females that had never been exposed to males and Control 2 included females housed with males for the entirety of their lifetimes. A ratio of 1 female to 5 males was used during the exposure treatments.

Females were individually introduced into vials containing the males as well as food medium. The females were exposed to the males for five hours. At the end of the exposure, females within a treatment were pooled back together into new vials. The number of living females in each vial was checked daily. Any dead female was removed from the treatment vial and the treatment, replicate number, and date of death were recorded. All surviving females were transferred to new vials every five days to avoid larval overcrowding and vial contamination. To control for the deterioration of females during anaesthetization, all replicate vials for all treatments and controls were exposed to CO_2_ as the start of each exposure. A minimum of 8 replicates per treatment was initiated.

### 2.4. Effect of Male Age on Mating Success

In our first experiment we observed that males outlived females to such an extent that it becomes possible for males to mate with females of successive generations. We therefore performed mate choice assays in* D. melanogaster*, to assess if aged males are competitive with younger males with respect to mating success. Newly emerged males and females were collected from the base population and maintained in vials at a density of 30–40 flies per vial with 50 : 50 ratio of males to females. Two days prior to the mating experiments, males were separated from females and housed individually in separate vials. In this manner we collected males aged 5, 10, 15, 20, and 25 days that had mating experience of 3, 8, 13, 18, and 23 days, respectively.

Virgin females from the base population were collected within 3 hours after eclosion and aged for five days for mating experiments. Pairs of 5-day-old versus 10-, 15-, 20-, and 25-day-old males were established. One male from each pair was marked with a notch on the wing to allow for identification. Even though wing clipping has not been found to interfere with mating success in* Drosophila* [49], we ensured that paired treatments within a set were reciprocally marked for half the treatments and tested for any significant effect of marking on male behavior. Each male pair (young and old) was introduced into a vial with a female. Trials were terminated if a successful copulation did not occur within 15 minutes. A trial was retained for statistical analysis only if both males courted the females.

In a separate experiment, we measured the number of progeny produced by 5-day-old males (mating experience of 3 days) versus 25-day-old males (mating experience of 20 days) in order to gain an idea of the differences in fitness contributions between young and old males. Each male was mated with 5-day-old virgin females. Following mating, females were removed and housed individually in a separate vial. Progeny emerging from each vial were counted for a period of 4 days. 25 replicates were done.

### 2.5. Statistical Analyses

All analyses were performed with R (R Development Core Team, 2006). An analysis of deviance (survival package for R) was conducted to test for the effects of treatments, sex, and species on longevity. Interactions between the different factors were also included using the following model: longevity ~ treatments + sex + species + treatments *∗* sex + treatment *∗* species + sex *∗* species + treatments *∗* sex *∗* species. We used the same model without the species factor to assess the effect of male exposure to female longevity. We used a chi square test with a single degree of freedom to test for deviation from random mating in the mating test experiments.

## 3. Results

### 3.1. Increasing Male Density Is Detrimental to Females; Males Are Unaffected across All Four Species

**Figure 1:**
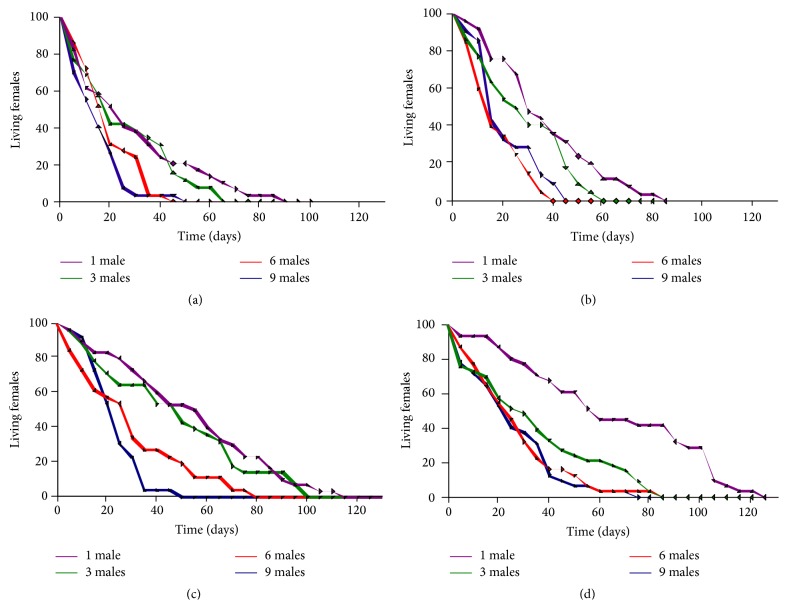
Proportion of surviving females exposed to an increasing density of males through time. (a)* D. melanogaster*. (b)* D. simulans*. (c)* D. sechellia*. (d)* D. mauritiana*.

**Figure 2:**
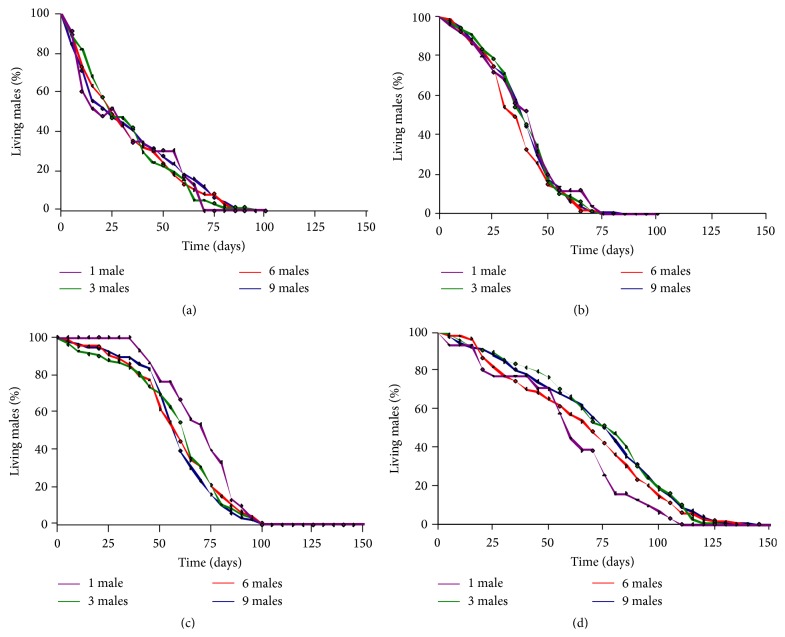
Proportion of surviving males exposed to an increasing density of males through time. (a)* D. melanogaster*. (b)* D. simulans*. (c)* D. sechellia*. (d)* D. mauritiana*.

In order to determine how increasing male/sex ratio (females exposed to increasing number of males) affects female fitness, we investigated the effect of increasing male density on both male and female viability (longevity) as well as on female fecundity across four closely related species of the* Drosophila melanogaster* subgroup (*D. melanogaster, D. simulans, D. mauritiana,* and* D. sechellia*). We found a strong difference in longevity between the sexes (*p* = 8.378 × 10^−43^) across species (*p* = 5.109 × 10^−141^; Figures 1 and 2, Table 1). We also observed significant interactions between treatments (male density) and sex (*p* = 5.068 × 10^−20^; Table 1) and between sex and species (*p* = 9.334 × 10^−7^), as well as between treatments, sex, and species (*p* = 8.888 × 10^−4^). Interestingly we found no significant interactions between species and treatment (*p* = 0.2). In summary, these results show that while all four species respond similarly to increasing male density, the sexes across species appear to respond differently.

We therefore separated the analysis of longevity of each sex in order to gain a better understanding of the effect of increased male density on the longevity of each sex. We found a significant treatment effect on female longevity; the greater the male density the shorter the female longevity across all four species (*p* = 3.497 × 10^−17^; Figure 1, Table 1).

In contrast, increasing male density did not affect male longevity at all (*p* = 0.2; Table 1). However, we observed a species effect on male longevity as* D. melanogaster* and* D. simulans* males have shorter average life expectancies than* D. mauritiana* or* D. sechellia* males (33.34 ± 24.64 days and 39.07 ± 16.29 days versus 70.26 ± 32.43 days and 60.09 ± 20.96 days, resp.). This may reflect natural variation or laboratory selection for shorter lifespans in the former species. In this experiment however no new males were added to the vials in order to replace old or dead males; it is therefore possible that the male/female ratio change across time could have affected female longevity. In order to test for such an effect, we performed the analysis of deviance again including the number of living males at female death within treatments; these tests did not alter our conclusions (*p* = 5.680 × 10^−16^ and *p* = 1.874 × 10^−11^, resp.).

**Figure 3:**
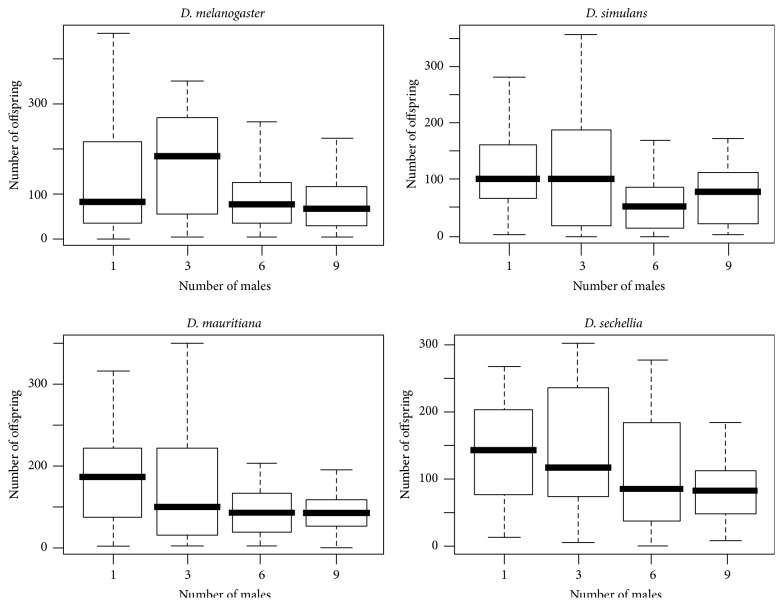
Number of offspring produced by females of* D. melanogaster, D. simulans, D. sechellia,* and* D. mauritiana* when exposed to increasing numbers of males.

Due to the significant relationship between longevity and the amount of offspring per female (*R* ^2^ = 0.503, *p* < 2 × 10^−16^), we used the residuals from the regression between longevity and number of offspring to test the effect of male density on offspring production. Using such a correction, we found no significant effect of male density on female fecundity (*F*_3,358_ = 1.7538, *p* = 0.1557) but we did observe a significant difference in female fecundity between species (*F*_3,358_ = 10.5014, *p* = 1.231 × 10^−6^). The difference here lies mainly in the slightly higher fecundity in* D. melanogaster* relative to the other three species (Figure 3). Differences between* D. simulans, D. mauritiana,* and* D. sechellia* were not significant.

### 3.2. Females Benefit from Multiple Matings, despite Reduction of Their Lifespan

**Figure 4:**
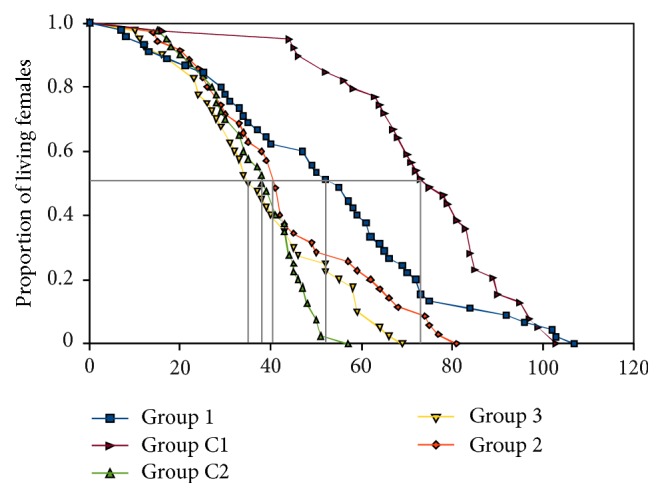
Comparison of female longevity when kept virgin (Group C1), females mated once in their lifetime (Group 1), females exposed to males every 7 days (Group 2), females exposed to males every three days (Group 3), and females kept with males for entire lifetime (Group C2).

We conducted further tests of the effect of male exposure on female longevity and fecundity in* D. melanogaster*. In this experiment, rather than housing females and males together continuously, we assessed the effect of the frequency/periods of male exposure on female fitness. Overall, we found a significant effect of treatment (*p* = 1.21 × 10^−14^; Figure 4) and no difference between replicates within treatments (*p* = 0.1167). After Bonferroni correction, we found that virgin females in Control 1 outlived mated females from all the other treatments as well as Control 2 (*p* < 0.05; Figure 4). In contrast, females exposed only once to males had a shorter lifespan than virgin females but lived significantly longer than females exposed once every three days to males, or compared to females continuously housed with males (*p* < 0.01 in all comparisons). There is, however, no difference between females exposed only once to males in their lifetime and females exposed to males once a week (*p* = 0.493; Figure 4). Overall, these results show that multiple mating decreases female lifespans significantly but the effect is not linear with the number of males courting the female.

**Figure 5:**
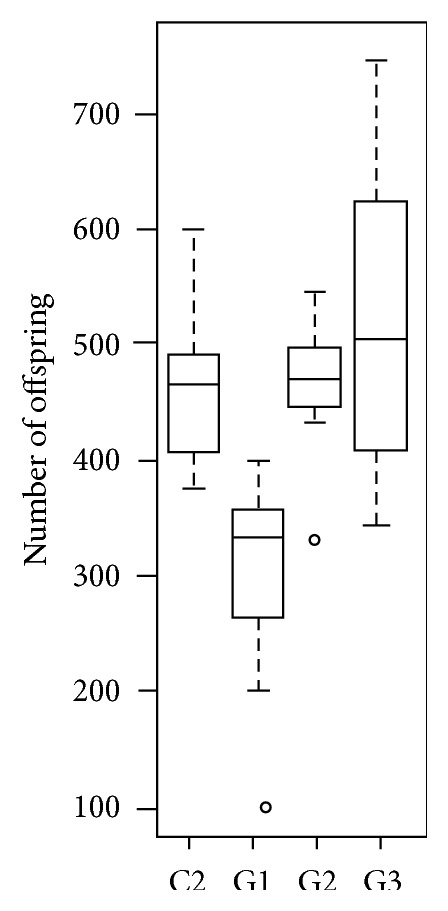
Comparison of the number of offspring between females, female kept with males for entire lifetime (Group C2), females mated once in their lifetime (G1), females exposed to males every 7 days (G2), and females exposed to males every three days (G3).

Although females mated once in their lifetime and never exposed to males afterwards live longer than females from most of the other treatments, we found a significantly lower number of offspring from this treatment compared to females with a greater exposure to males (treatments 2 and 3, *p* = 0.02162 and *p* = 0.02334, resp., after Bonferroni correction) and females housed with males (control 2, *p* = 0.01246 after Bonferroni correction, Figure 5).

### 3.3. Older Males Are Competitive against Younger Males in Remating

In the first experiment above we observed that under conditions of biased sex ratio males outlived females to the extent that it raised the question of the propensity of males to mate with females from successive generations as they age. In order to test this hypothesis we performed mating competition assays in* D. melanogaster*, between 10-, 15-, 20-, and 25-day-old mated males and 5-day-old virgin males.

Within each group we found no significant difference in mating success between 5-day-old males versus 10- (*p* = 0.8, *n* = 60), 15- (*p* = 0.1, *n* = 65), 20- (*p* = 0.2, *n* = 62), or 25-day-old (*p* = 0.7, *n* = 61) males. Males were marked with notched wings and of the 248 matings scored, 113 successful males had clipped wings and 135 were with nonclipped wings. These differences are not statistically significant (*p* = 0.16) according to Fisher's exact tests, confirming that notching had no significant effect on mating success.

We also looked for any difference in the number of offspring produced by younger males (5 days old) or older males (25 days old), as a measure of their potential fitness contributions. Although younger males produced, on average, a higher number of offspring compared to older males the latter did quite well (*p* = 0.013; mean ± 95% CI: young = 312.8 ± 21.1, old = 228.7 ± 14.3, *n* = 25).

## 4. Discussion

One of the consequences of male mating strategies in* D. melanogaster* is the reduction of female lifespan, either due to harassment during courtship [22], or physical trauma during mating [13], or as a result of toxic seminal fluid proteins transferred during copulation [50]. Whether or not these sex specific fitness consequences are common across* Drosophila* species in a phylogenetic context has not been tested. Such studies are useful to test the generality of any condition across species in a taxonomic group. Our results show that sex specific fitness costs of mating are common and similar across species of the* D. melanogaster, D. simulans* clade; females suffer lifespan reduction costs; males are unaffected.

While the evolution of such antagonistic traits has been attributed to female choice or sexual conflict [3, 15, 51], little has been discussed with respect to the consequences of unequal lifespans of the sexes, on net population fitness (but see [52, 53]). This issue may be relevant to determining whether or not sexually antagonistic coevolution will take place [17, 54]. Our assays conducted with* D. melanogaster* suggest that, despite a reduction in their lifespan, females do benefit from a certain number of increased matings. Quite importantly, our results suggest that male reproductive longevity provides opportunities for additional gains of fitness by males, which can benefit populations.

Darwin had noted that males, in a variety of animals [55], are almost always the active seekers and initiators of sexual interactions, using a variety of means, from song and dance, to coercion. These male behaviors are likely to influence the manner in which females respond to male sexual behaviors, thereby affecting the pattern, intensity, and direction of sexual selection or conflict in populations. Indeed, recent research focusing on male life history [56, 57] is beginning to shed more light on the interrelationship between life history and sexual selection/conflict.* Drosophila melanogaster* males seem to exercise choice by adjusting their ejaculate size based on female status [58]; they also adjust the nature of ejaculate to manipulate female behaviour and physiology [59, 60] and to compete with rival males [61]. Such studies and our present study exploring the role of male life history will be important in testing traditional ideas that males are indiscriminate and tend to “live fast and die young” [62, 63] and the assumption of the twofold costs of sex [64] that males typically do not contribute more than their gametes.

Below, we examine our results in the perspective of male driven sexual selection (see [65, 66]) to highlight that not only is the fitness differential between sexes in lifetime reproductive success a male driven phenomenon but also the additional gains of lifetime reproductive fitness by males might render the loss of female lifetime reproductive fitness to evolve as a nearly neutral trait, preventing the expression of sexually antagonistic coevolution.

### 4.1. Increasing Exposure to Males Reduces Female Longevity but Not Male Longevity

Our results extend what has previously been shown in* D. melanogaster* [23, 67–69], and in* D. simulans* [35], to be common across all four related species of the* melanogaster-simulans* clade. This result is important in showing that, despite radically different ecologies and life histories, the mating strategies and costs of mating to females are similar (Figure 1).* D. melanogaster* and* D. simulans* are cosmopolitan and sympatric.* D. mauritiana* and* D. sechellia* are island endemics (to Mauritius and Seychelles, resp.). Amongst the four species,* D. sechellia* has a distinctly different life history by having specific ecological and physiological adaptations to the* Morinda citrifolia* plant [46]. Compounds found in the fruit of this plant are toxic to the other three species [46]. Apart from longevity differences between* D. melanogaster* and* D. simulans* compared to* D. mauritiana* or* D. sechellia* (see Section 3) we found no major differences on mating outcomes of the sexes; female longevity across species is significantly reduced with increasing and continuous exposures to males. However, intermittent exposure to fewer males was found to be less harmful to* D. simulans* female lifespan (Figure 1, also see [35]) as well as for* D. melanogaster* (this study). In fact, it is quite interesting that the results from assays using* D. melanogaster* alone (Figure 4, see below) and Taylor et al.'s [35] study in* D. simulans* suggest that the optimal number of males and matings that is least harmful to female lifespan and maximizes female fitness (progeny produced) in these cosmopolitan species appears to be three (Figures 4 and 5). Females exposed to six or nine males suffered greater reduction of lifespan and produced less progeny. It is also quite interesting to see that the two cosmopolitan species appear to benefit somewhat from polyandry, in terms of fecundity (Figure 3), but this is not true for the island endemics that appear to benefit more from monogamy (Figure 3). These differences should however be treated with some caution because* D. melanogaster* was an outbred population compared to the isofemale lines of the members of the* simulans* complex (see Section 2). Further research, particularly in wild populations, will be useful in validating our results and speculations regarding species differences in fecundity.

On the other hand our results show that increasing male density has no negative effects on male longevity across all species (Figure 2). Direct male-male interaction may be an important determinant of sexually selected traits in* Drosophila* males and it may be expected that intense male-male competition is more likely in high male density conditions and is therefore detrimental to males (e.g., see [70]). The lack of such a result is interestingly viewed in the light that male-male competition in* Drosophila* may largely occur via sperm competition within the female's reproductive tract [4, 30, 69]. One of the consequences of this male-specific evolution is the reduction of female lifespan due to toxicity of some seminal fluids [71].

### 4.2. Females Benefit from Multiple Matings despite a Reduction of Their Lifespan

Assays done in* D. melanogaster* to study the effect of temporal exposure of females to males show that the optimum number of matings in terms of number of offspring produced is greater than one. Females mated only once produce a lower number of offspring compared to females exposed to males once a week, even though females in both treatments have comparable life expectancies (Figure 5). These results are consistent with several other studies across insects, which indicate that, despite numerous deleterious effects that may be linked to remating, there are nonetheless many direct and indirect advantages to female fitness [37, 38, 72–74]. Females can benefit from remating through nuptial gifts, increased postcopulatory feeding, and increased resistance to starvation and desiccation which can in some cases outweigh male induced harm [75–77]. Even experimentally induced harm did not deter female remating propensity in several insects [78]. In addition, it is noteworthy that male seminal fluids influence females ovipositional behavior and physiology in* D. melanogaster*, which may contribute indirectly to increasing female fitness despite the costs to lifespan [50]. These results may warrant a speculation that difference in fitness optima between the sexes, with respect to lifetime reproductive success, may not be sufficiently large enough for natural selection to act against it, such that sexually antagonistic coevolution may not take effect. However, it is important to note that our results of a narrow range of matings (~3) that minimizes this fitness differential (Figure 5) may be an underestimation, perhaps typical of laboratory conditions. Studies in natural populations of* D. melanogaster* using microsatellite markers to determine paternity of progeny from females captured in the wild reveal that four to six remating occurrences may be more typical in the wild [79]. In addition, there is some evidence that mated females may live longer than virgins in nature [80]. This may be, as Taylor et al. [35] surmised, because females in nature are not continuously exposed to males, as in a laboratory setting. More studies in the typical number of matings that occur for males and females in natural populations will be needed to shed additional light on this issue.

### 4.3. Longer Male Lifespan, Transgenerational Mating, and Male Gains of Fitness

Not only do our results show longer life expectancy of males in all species (Figure 2), but, further exploring the relevance of this result in* D. melanogaster*, we found that males as old as 25 days have the similar probability of mating success compared with much younger males. Longer male reproductive lifespan is quite interesting in the context of some other factors, which bring to light the reproductive potential of older males and their potential to contribute positively to population fitness. Some lines of evidence suggest the propensity of older males to mate with younger females in* Drosophila*. In the wild, males have been observed to patrol pupal sites in order to mate with teneral females [9]. Older males may benefit from the fact that younger* D. melanogaster* females are either less choosy or are not as efficient in rejection behaviors as older females [81, 82]. In fact, Saleem et al. [83] have shown that older males typically outcompete younger males in courtship and mating. In* D. bipectinata,* females prefer to mate with older males [84]. All of these data, along with our results of comparable mating success between young and older males, suggest the potential of males to mate with females of different generations. However, Edward and Chapman [57] found that the number of offspring sired by males declines with age. This is also true in our study; younger males sired, on average, more offspring. However, males as old as 25 days are capable of producing offspring, and since they are likely to have mated (perhaps several times) in their earlier days, even a single or a couple of extra matings count as added contributions to population fitness. Therefore, even if females typically die early as a result of mating (or multiple mating), older males can mate with younger females, many of whom are likely to belong to successive generations. This creates a bias in the effective population size of males relative to females, which can increase male reproductive variance relative to that of the females.

Indeed, observations on natural populations suggest the existence of such a reproductive asymmetry in nature. There is a well-described excess of polymorphism on the X chromosome relative to the autosomes among African populations [67, 85, 86]. Aside from the effects of population expansion, which are less likely for the ancestral African populations [87, 88], increased reproductive variance of males relative to the females is the main explanation for such imbalance [89, 90]. Our observations were made in controlled laboratory conditions and experiments simulating natural conditions need to be performed; it is unlikely that the extent of female death in closed laboratory settings can be extended to nature, where females have ample opportunity to escape males. On the same note, it is also intriguing that selection against female death has not operated on laboratory* Drosophila* populations. It is certainly not true that all mated females suffer similar mortality rates; that is, there is genetic variation underlying the variation in female mortality. This variation represents a substrate that natural selection can operate on to select against factors reducing female fitness.

Intergenerational mating creates a unique situation that can offset at least some if not all costs due to early female mortality; even though females' lifespan is reduced due to mating, there are younger females that males can mate with. As a result, loss of fitness due to deaths of mated females will be inconsequential in the scheme of natural selection. This situation can have broad implications on the life expectancy of the sexes (see [52, 53]). For instance, Bonduriansky [52] has suggested that female life expectancy may play a major role in how the sexes coevolve, based on the intensity of sexual conflict and extrinsic factors that may affect female mortality. For instance, in high female mortality conditions (harsh environments or predation), females stand to benefit from male mating strategies, including those that are costly to them. On the other hand, favorable environments can result in more resistant females [52]. Our study would imply that male mating strategy is also a factor that shapes female life expectancy, and early female demise obstructs the ability for females to evolve counter adaptations to male's harmful effects. This would fit a prediction where the potential for sexually antagonistic coevolution may exist, but it is not realized [17]. This would be an important factor to consider in continuously mating populations. Indeed, reproductive longevity of older males and preference for younger females have been suggested to have contributed to the increase in longevity in humans [91]. The result of older male contributions will require further detailed study due to the broad implications of reproductive longevity of males and shortened life expectancy of females.

## 5. Conclusions

All else being equal, the Fisherian principle of sex ratio evolution would in theory predict that natural selection should favor a sexual system where both sexes contribute equally in every generation [92]; however everything is generally not equal in sexual organisms. Our study addresses the fitness inequality between sexes that arises as a result of male-female competition in mating/remating and provides a possible explanation as to how many additional fitness contributions of males may be able to compensate or at least minimize the fitness lost due to early female deaths. As a result the fitness differential between sexes may not be sufficiently large enough to create a load on populations such that natural selection will act against it. The sexual disparity in fitness caused by individual male-female competition, wherever it occurs, would not matter as long as population fitness is not affected. Additional studies on the relevance of male reproductive longevity and female benefits of remating will be useful in further tests of our proposition.

## Figures and Tables

**Table 1:** Analysis of deviance on individual longevity from experiments 1 and 3 testing the effects of male density (treatments) on female and male longevity (sex), across *D*. *melanogaster*, *D*. *simulans*, *D*. *sechellia,* and *D*. *mauritiana*.

	df	Deviance	*p* value
Male density	3	4.9	0.2
Sex	1	188.1	8.378 × 10^−43^
Species	3	652.1	5.109 × 10^−141^
Male density × sex	3	93.0	5.068 × 10^−20^
Male density × species	9	13.1	0.2
Sex × species	3	30.8	9.334 × 10^−7^
Male density × sex × species	9	28.2	8.888 × 10^−4^
Females			
Male density	3	79.7	3.497 × 10^−17^
Species	3	45.1	8.656 × 10^−10^
Male density × species	9	16.1	0.1
Males			
Male density	3	4.5	0.2
Species	3	664.2	1.234 × 10^−143^
Male density × species	9	15.8	0.1
